# Apremilast as a novel therapeutic option for psoriasis coexisting primary sclerosing cholangitis and ulcerative colitis: A case report

**DOI:** 10.1097/MD.0000000000045232

**Published:** 2025-10-24

**Authors:** Jingya Gao, Yue Xiao, Ping Tan, Hongxiang Hu, Wenyao Mi, Yiyi Wang, Wei Li

**Affiliations:** a Department of Dermatology & Venerology, Rare Disease Center, West China Hospital, Sichuan University, Chengdu, China.

**Keywords:** apremilast, case report, primary sclerosing cholangitis, psoriasis, ulcerative colitis

## Abstract

**Rationale:**

Plaque psoriasis, primary sclerosing cholangitis, and ulcerative colitis are all immune-mediated inflammatory diseases. Their coexistence is extremely rare, and therapeutic management remains challenging due to shared immunopathogenic mechanisms and limited treatment experience.

**Patient concerns:**

A 36-year-old woman presented with pruritic red papules and plaques covering scales all over the body for more than 10 years. She also reported liver dysfunction for over 3 years and chronic diarrhea exceeding 1 year.

**Diagnoses:**

Based on her medical history, clinical manifestations, histopathological findings, and examinations, she was diagnosed with plaque psoriasis, primary sclerosing cholangitis, and ulcerative colitis.

**Interventions:**

The patient had shown an inadequate response to oral steroids and adalimumab. Upon presentation to our clinic, she was assessed as having moderate plaque psoriasis (Psoriasis Area and Severity Index: 6.0, body surface area: 10.0%) and was treated with oral apremilast (titrated up to 60 mg/d), ursodeoxycholic acid (1000 mg/d), and phosphatidylcholine (1368 mg/d).

**Outcomes:**

After 48 weeks of treatment, the patient achieved complete clearance of psoriatic lesions, marked reduction of pruritus, stable liver function, decreased diarrhea frequency, and no adverse events.

**Lessons:**

This case underscores the potential response and tolerability of apremilast as an alternative treatment for psoriasis coexisting with autoimmune liver disease and inflammatory bowel disease.

## 1. Introduction

Psoriasis is an immune-mediated, inflammatory disease manifested as scaly erythematous plaques, limited or widely distributed, with a global prevalence of 0.1% to 1.5%.^[1]^ Comorbidities triggered by systemic inflammation occurring more commonly in psoriasis patients, such as psoriatic arthritis, metabolic syndrome, cardiovascular disease, and inflammatory bowel disease (IBD) in which the association (odds ratio) with psoriasis is 2.0 (95% confidence interval: 1.4–2.9) for Crohn’s disease and 1.5 (95% confidence interval: 1.2–2.0) for ulcerative colitis (UC).^[2]^ Primary sclerosing cholangitis (PSC) is a rare chronic cholestatic liver disease characterized by fibroinflammatory damage to the biliary tree, classified as an immune-mediated inflammatory disease (IMID). The diagnosis is based on the presence of elevation of cholestatic indices and imaging findings, with liver biopsy being nonessential. Its clinical symptoms like pruritus and fatigue are often nonspecific, and there is currently no effective pharmacological therapy available.^[3]^ Current clinical evidence suggests that UC and PSC are frequently comorbid and that there is a high correlation between psoriasis and UC.^[4,5]^ However, the coexistence of PSC, UC, and psoriasis in the same patient is rare, and choosing the effective treatment for these complicated conditions can be challenging. As known, apremilast is a practical, well-tolerated, and convenient option for treating psoriasis and psoriatic arthritis.^[6,7]^ In another study, apremilast also attenuated the clinical features of UC.^[8]^ Here, we report a case of psoriasis combined with PSC and UC treated with apremilast, which showed significant improvement in skin lesions and diarrhea symptoms.

## 2. Case description

A 36-year-old female with a >10-year history of generalized scaly erythema, with abnormal liver function for >3 years and diarrhea for >1 year, was referred to our department. The patient presented with pruritic red papules and plaques covering scales all over the body for >10 years. She had been treated with multiple topical medications to alleviate psoriatic skin lesions. Then she was hospitalized for a liver dysfunction with jaundice and bilirubinuria developed with hepatoprotective therapy in the local hospital 3 years ago and continued maintenance therapy with ursodeoxycholic acid (UDCA) 500 mg and ademetionine 1000 mg daily. At the same time, her scaly erythema appeared explosively with less effect of topical medications and phototherapy. One year before, diarrhea and abdominal pain occurred and her liver function continued to fail to improve; her stools were irregular, and about 7 times a day without watery like. She had a colon biopsy and was treated with UDCA 1000 mg, azathioprine 50 mg, and methylprednisolone 32 mg daily due to a possible diagnosis of PSC and UC at the gastroenterology department in our hospital. Due to the uncontrolled liver dysfunction, the patient was prescribed hydrocortisone for 3 months, which was discontinued because of osteoporosis. A maintenance therapeutic regime (UDCA 1000 mg/day, phosphatidylcholine 1368 mg/day) has been used after cessation of steroids. After a comprehensive biologic agent screening, she received a 4-week course of adalimumab under the guidance of the gastroenterology department but discontinued it due to poor efficacy. However, her scaly erythema and diarrhea were without improvement, and she was recommended to our clinic.

**Figure 1.**
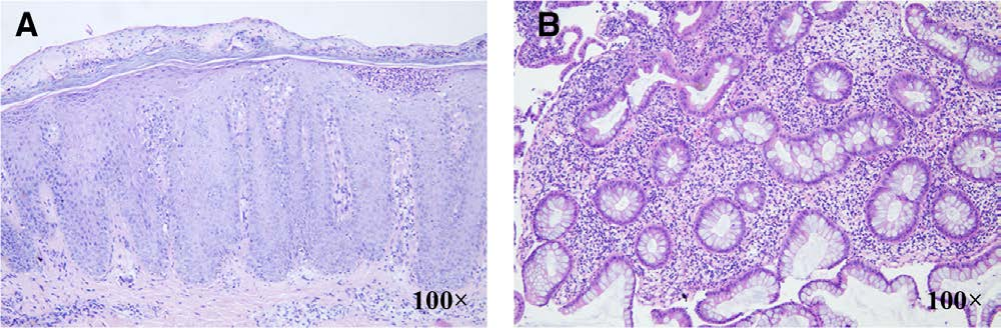
(A) Histopathology of the lesion on the abdomen skin revealed hyperkeratosis, parakeratosis, acanthosis in the epidermis, neutrophil aggregates forming Munro-micro abscesses, dermal papilla uplift, capillary lumen dilation with infiltration of lymphocytes in the dermis. (B) Histological analysis of the patient’s transverse colon biopsy revealed more chronic inflammatory cell infiltration in the mucosa, with crypt branching and distortion.

Physical examination revealed scattered red papules and plaques with scales on the patient’s lower limbs, arms, lower back, and scalp along with a typical Auspitz sign. The psoriasis area severity index is 6.0, and the body surface area is 10.0%. Laboratory examination revealed abnormal liver function and cholestasis with high total bilirubin (53.0 μmol/L; reference range 5.0–28.0 μmol/L); alanine aminotransferase: 130 IU/L (reference range < 40 IU/mL); aspartate aminotransferase: 126 IU/L (reference range < 35 IU/mL); alkaline phosphatase: 469 IU/L (reference range 35–100 IU/mL); glutamyl transpeptidase: 136 IU/L (reference range < 45 IU/mL). The patient tested negative for hepatitis B antigen and antibody. The C-reactive protein and erythrocyte sedimentation rate and immunoglobulins, including IgG, IgA, and IgM, were elevated. No abnormalities were detected in other examinations. Histopathology of the abdominal skin and transverse colon were consistent with psoriasis and UC, respectively, serving as the gold standard for diagnosis (Fig. 1). MRI of the upper abdomen during hospitalization showed mild liver enlargement, thickening and strengthening of the gallbladder wall, mild dilatation of the intrahepatic bile ducts and common hepatic ducts, and localized luminal narrowing. After excluding secondary cholestatic liver diseases, significant imaging findings and elevated alkaline phosphatase and glutamyl transpeptidase support the diagnosis of PSC. The patient received Apremilast in addition to her ongoing therapy with UDCA (1000 mg/day) and phosphatidylcholine (1368 mg/day) for 48 weeks. Apremilast was initiated at 10 mg and titrated up to 30 mg twice daily, while the existing medications remained unchanged.

**Figure 2.**
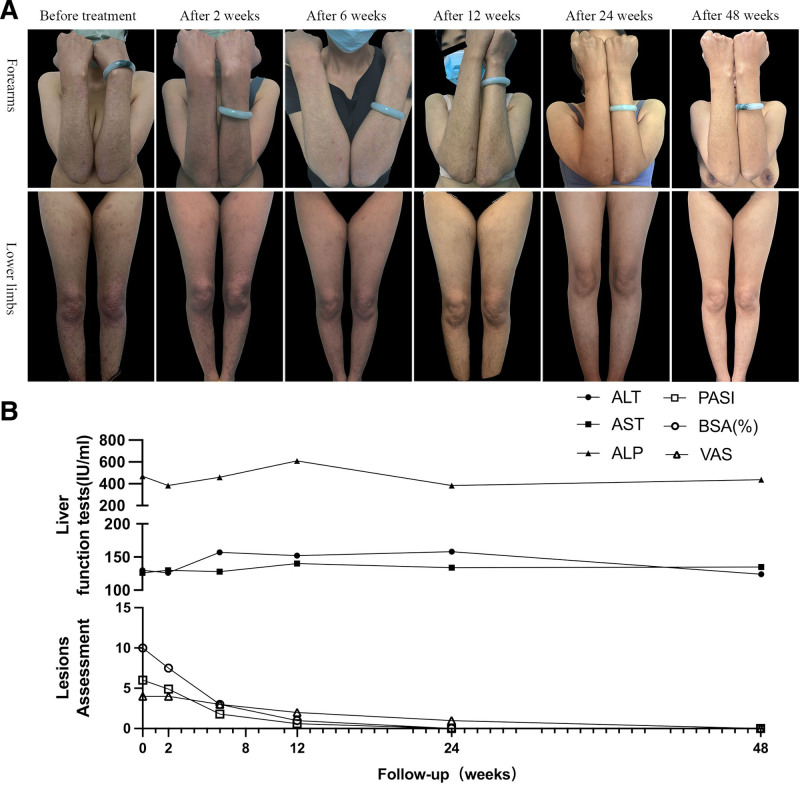
(A) Clinical features of psoriatic lesions before and after apremilast treatment. (B) The psoriasis severity was assessed using the psoriasis area severity index (PASI) and body surface area (BSA). Pruritus severity was evaluated through the visual analog scale (VAS), with scores ranging from 0 to 10. The patient attained a maximum PASI score of 6.0 and a BSA of 10.0%. Consequently, the lower y-axis extends to 15. The line graph illustrates remission in PASI, BSA, and VAS over the 48 weeks. The upper *y*-axis for liver function tests displays scores ranging from 100 to 800, with stabilized results. ALT = alanine aminotransferase, ALP = alkaline phosphatase, AST = aspartate aminotransferase.

The patient reported that the pruritus was significantly reduced, reflected on the visual analog scale score and the skin lesions gradually darkened and faded (Fig. 2A). After 48 weeks of treatment, she achieved complete healing of erythematous scales and diarrhea with psoriasis area severity index and body surface area scores of 0. During follow-up, C-reactive protein and erythrocyte sedimentation rate remained slightly above normal, liver function remained stable (Fig. 2B), the frequency of diarrhea was reduced, and no gastrointestinal adverse events, infections, or other complications were observed. Although the patient remained on UDCA and phosphatidylcholine, the improvements in skin lesions and diarrhea after initiation of apremilast suggest that it was likely the main contributing treatment.

## 3. Discussion

Herein, we report a case of psoriasis combined with PSC and UC successfully treated with apremilast. The patient exhibited complete resolution of skin lesions, amelioration of diarrhea symptoms, and stabilized liver function after treatment. Notably, there is no report on the utilization of apremilast in psoriasis coexistence of PSC and UC.

Psoriasis, PSC, and UC are three IMIDs sharing potential common pathogenic risk loci.^[9]^ Psoriasis and IBD have a high risk of comorbidity, possess substantial genetic predisposition, and involve the increased expression of the cytokines interleukin 17 (IL-17), IL-23, and tumor necrosis factor-α (TNF-α).^[2,10]^ Meanwhile, PSC demonstrates a close association with IBD, affecting up to 80% of PSC patients.^[4]^ A prospective cohort of 195 PSC patients revealed a 3.6% prevalence of psoriasis, similar to the local general population, indicating no significant correlation between psoriasis and PSC.^[11]^

Moreover, the management of patients with multiple comorbidities is challenging, and an individualized therapeutic scheme is needed. Given the fluctuating liver function indexes in this patient, the use of traditional disease-modifying antirheumatic drugs, such as methotrexate with significant hepatotoxicity, should be avoided. Additionally, despite TNF-α inhibitors being indicated for psoriasis and UC, the patient’s prior inadequate response to adalimumab, coupled with evidence suggesting that TNF-α therapy may exacerbate psoriatic skin lesions in the context of IBD, renders this treatment option less favorable.^[12]^ Due to contraindications in IBD, IL-17 inhibitors were excluded. The use of IL-23 inhibitors is currently limited to case report which may restrict the application of targeted therapy.^[13]^ Previous studies on JAK inhibitors for the treatment of IBD with concurrent PSC reported adverse reactions in 17% of patients, indicating potential risks.^[14]^ Given the presence of multiple IMIDs in this patient, multiple cytokine patterns are simultaneously activated and are likely pathogenically relevant at the sites of IMIDs. Therefore, combination therapy, using one or more drugs targeting different inflammatory pathways, may be a better option. PDE-4 inhibitors represent a potential exploratory treatment option.

Apremilast, a selective inhibitor of PDE4-cAMP signaling, represents an innovative therapeutic strategy for psoriasis in cases where other systemic therapies have failed or been contraindicated.^[15]^ Growing evidence shows that patients with inflammatory diseases exhibit higher PDE-4 expression than healthy individuals, including but not limited to rheumatoid arthritis, chronic obstructive pulmonary disease, and autoimmune disease.^[16]^ Currently, PDE-4 inhibitors show substantial potential in the treatment of IBD. PDE-4, in particular, is expressed in dendritic cells, macrophages, monocytes, and T cells, and is considered an important player in the inflammatory response. Elevated intracellular cAMP levels were detected in UC patients, leading to downregulation of the inflammatory response in the mucosa (NF-κB, TNF-α, IL-1β, IL-17, IL-23 etc).^[17,18]^ Based upon this, some specialists suggest apremilast as a third-line option for psoriasis patients with comorbid UC.^[19]^ Although a previous study demonstrated similar levels of PDE-4 in PSC patients compared to the healthy subjects, PDE-4 is considered to play a role in attenuating fibrogenic signaling in a bile-duct ligation liver fibrosis rat model.^[20,21]^

The overall safety profile of apremilast is favorable, with reported adverse events such as diarrhea, nausea, and vomiting being primarily mild to moderate and often alleviated within 2 to 4 weeks.^[15]^ In this case, the patient did not suffer such adverse effects, and the diarrhea symptoms improved after twelve weeks of apremilast treatment. In addition, clinical trials of apremilast have shown encouraging results without liver abnormalities in both short- and long-term exposures.^[22,23]^

## 4. Conclusion

This case underscores the potential response and tolerability of apremilast as an alternative option for treating psoriasis combined with autoimmune liver disease and IBD. However, further evidence is warranted in the future.

## Author contributions

**Conceptualization:** Jingya Gao, Wei Li.

**Data curation:** Ping Tan, Hongxiang Hu.

**Formal analysis:** Wenyao Mi, Yiyi Wang.

**Writing – original draft:** Jingya Gao, Yue Xiao.

**Writing – review & editing:** Yue Xiao, Wei Li.
